# Diminished Posterior Precuneus Connectivity with the Default Mode Network Differentiates Normal Aging from Alzheimer's Disease

**DOI:** 10.3389/fnagi.2017.00097

**Published:** 2017-04-19

**Authors:** Bernadet L. Klaassens, Joop M. A. van Gerven, Jeroen van der Grond, Frank de Vos, Christiane Möller, Serge A. R. B. Rombouts

**Affiliations:** ^1^Institute of Psychology, Leiden UniversityLeiden, Netherlands; ^2^Department of Radiology, Leiden University Medical CenterLeiden, Netherlands; ^3^Leiden Institute for Brain and Cognition, Leiden UniversityLeiden, Netherlands; ^4^Centre for Human Drug ResearchLeiden, Netherlands

**Keywords:** Alzheimer's disease, dementia, aging, brain connectivity, functional network, resting state fMRI, functional connectivity

## Abstract

Both normal aging and Alzheimer's disease (AD) have been associated with a reduction in functional brain connectivity. It is unknown how connectivity patterns due to aging and AD compare. Here, we investigate functional brain connectivity in 12 young adults (mean age 22.8 ± 2.8), 12 older adults (mean age 73.1 ± 5.2) and 12 AD patients (mean age 74.0 ± 5.2; mean MMSE 22.3 ± 2.5). Participants were scanned during 6 different sessions with resting state functional magnetic resonance imaging (RS-fMRI), resulting in 72 scans per group. Voxelwise connectivity with 10 functional networks was compared between groups (*p* < 0.05, corrected). Normal aging was characterized by widespread decreases in connectivity with multiple brain networks, whereas AD only affected connectivity between the default mode network (DMN) and precuneus. The preponderance of effects was associated with regional gray matter volume. Our findings indicate that aging has a major effect on functional brain interactions throughout the entire brain, whereas AD is distinguished by additional diminished posterior DMN-precuneus coherence.

## Introduction

When age progresses, the brain is subjected to many changes that are related to deterioration of sensory, motor and intellectual functioning (Salthouse, 1996; Li and Lindenberger, 2002; Fandakova et al., 2014). In Alzheimer's disease (AD), a gradual worsening in memory and other cognitive domains occurs, accompanied by a notable reduction in independency and daily life functioning (McKhann et al., 2011). This age and dementia related decline in function is likely to be associated with a loss of integrity of large-scale brain networks (Mesulam, 1998). Accordingly, functional network connectivity as measured with functional magnetic resonance imaging (fMRI) is diminished in normal aging and AD (Sperling, 2011; Hafkemeijer et al., 2012; Ferreira and Busatto, 2013; Barkhof et al., 2014; Betzel et al., 2014; Sala-Llonch et al., 2015).

The default mode network (DMN) has been preferentially studied, as its core regions (precuneus, posterior cingulate cortex) are relevant for episodic memory retrieval (Greicius et al., 2004; Lundstrom et al., 2005) and susceptible to accumulation of β-amyloid (Buckner et al., 2005) in older adults and patients with AD. Both aging and AD are most prominently characterized by a reduction in DMN connectivity (Greicius et al., 2004; Damoiseaux et al., 2008; Biswal et al., 2010; Koch et al., 2010; Zhang et al., 2010; Pievani et al., 2011; Hafkemeijer et al., 2012; Ferreira and Busatto, 2013; Dennis and Thompson, 2014).

There are also indications for connectivity change in other brain networks in aging (Andrews-Hanna et al., 2007; Wu et al., 2007a,b; Allen et al., 2011; Yan et al., 2011; Mowinckel et al., 2012; Onoda et al., 2012; Tomasi and Volkow, 2012) and AD (Zhou et al., 2010; Agosta et al., 2012; Binnewijzend et al., 2012; Brier et al., 2012; Sheline and Raichle, 2013). However, this has been studied less well and results tend to be mixed. For example, contradicting results have been found for the visual system in older adults (Andrews-Hanna et al., 2007; Allen et al., 2011; Yan et al., 2011; Mowinckel et al., 2012; Onoda et al., 2012).

Although previous work suggests overlap and differences in functional connectivity patterns in normal aging and AD, it has not yet been investigated how changes due to older age relate to changes as seen in AD. Here, we compare voxelwise connectivity between young and older adults and between older adults and patients with AD with 10 standard functional networks as obtained by imaging 36 subjects at rest (Smith et al., 2009). Since aging and AD are primarily characterized by gray matter atrophy (Sluimer et al., 2009), it is encouraged to evaluate whether group differences in connectivity are explained by underlying gray matter loss (Oakes et al., 2007). We therefore present our results with and without correction for regional gray matter volume.

## Methods

### Subjects and design

We included 12 young subjects, 12 older adults, and 12 AD patients in this single center study (see Table 1 for demographics and Supplementary Figure 1 for additional background information on cognitive performance on the computerized NeuroCart® test battery). The clinical diagnosis of probable AD was established according to the revised criteria of the National Institute of Neurological and Communicative Disorders and Stroke and the Alzheimer's Disease and Related Disorders Association (NINCDS-ADRDA) (McKhann et al., 2011), including clinical and neuropsychological assessment. All AD patients participating in this study were recently diagnosed and had mild to moderate cognitive deficits with a Mini Mental State Examination (MMSE) score of at least 18 (Burns, 1998). Furthermore, they were assessed by a physician (i.e., neurologist, geriatrist) as mentally capable of understanding the implications of study participation.

**Table 1:** **Demographics of young and older adults and AD patients**.

	**Young adults**	**Older adults**	**AD patients**
*N*	12	12	12
Age (mean ± SD)	22.8 ± 2.8	73.1 ± 5.2	74.0 ± 5.2
Age range	18–27	64–79	65–81
Male/female	6/6	6/6	6/6
MMSE (mean ± SD)	29.9 ± 0.3	29.3 ± 0.9	22.3 ± 2.5

All subjects underwent a thorough medical screening to investigate whether they met the inclusion and exclusion criteria. They had a normal history of physical health and were able to refrain from using nicotine and caffeine during study days. Exclusion criteria included positive drug or alcohol screen on study days, regular excessive consumption of alcohol (>4 units/day), caffeine (>6 units/day) or cigarettes (>5 cigarettes/day) and use of benzodiazepines, selective serotonin reuptake inhibitors, cholinesterase inhibitors, monoamine oxidase inhibitors or other medication that is likely to alter resting state connectivity. The study was approved by the medical ethics committee of the Leiden University Medical Center (LUMC). Written informed consent was obtained from each subject prior to study participation. To compensate for the small sample sizes and increase the statistical power, six resting state fMRI (RS-fMRI) scans were analyzed per subject, giving 72 RS-fMRI scan series per group. Subjects were scanned two times (with 1 h in between) on three different occasions within 2 weeks. These data concern the baseline measurements that were acquired as part of a project in which the same subjects were measured before and after an intervention. The results of this intervention study will be published elsewhere.

### Imaging

“Scanning was performed at the LUMC on a Philips 3.0 Tesla Achieva MRI scanner (Philips Medical System, Best, The Netherlands) using a 32-channel head coil. During the RS-fMRI scans, all subjects were asked to close their eyes while staying awake. They were also instructed not to move their head during the scan. Instructions were given prior to each scan on all study days. T1-weighted anatomical images were acquired once per visit. To facilitate registration to the anatomical image, each RS-fMRI scan was followed by a high-resolution T2^*^-weighted echo-planar scan. Duration was approximately 8 min for the RS-fMRI scan, 5 min for the anatomical scan and 30 s for the high-resolution scan.

RS-fMRI data were obtained with T2^*^-weighted echo-planar imaging (EPI) with the following scan parameters: 220 whole brain volumes, repetition time (TR) = 2,180 ms; echo time (TE) = 30 ms; flip angle = 85°; field-of-view (FOV) = 220 × 220 × 130 mm; in-plane voxel resolution = 3.44 × 3.44 mm, slice thickness = 3.44 mm, including 10% interslice gap. The next parameters were used to collect T1-weighted anatomical images: TR = 9.1 ms; TE = 4.6 ms; flip angle = 8°; FOV = 224 × 177 × 168 mm; in-plane voxel resolution = 1.17 × 1.17 mm; slice thickness = 1.2 mm. Parameters of high-resolution T2^*^-weighted EPI scans were set to: TR = 2,200 ms; TE = 30 ms; flip angle = 80°; FOV = 220 × 220 × 168 mm; in-plane voxel resolution = 1.96 × 1.96 mm; slice thickness = 2.0 mm (Klaassens et al., 2017, p. 311).”

#### Functional connectivity analysis

##### Data preprocessing

All analyses were performed using the Functional Magnetic Resonance Imaging of the Brain (FMRIB) Software Library (FSL, Oxford, United Kingdom) version 5.0.7 (Smith et al., 2004; Woolrich et al., 2009; Jenkinson et al., 2012). “Each individual functional EPI image was inspected, brain-extracted and corrected for geometrical displacements due to head movement with linear (affine) image registration (Smith, 2002). Images were spatially smoothed with a 6 mm full-width half-maximum Gaussian kernel. Registration parameters for non-smoothed data were estimated to transform fMRI scans into standard space and co-registered with the brain extracted high resolution T2^*^-weighted EPI scans (with 6 degrees of freedom) and T1 weighted images (using the Boundary-Based-Registration method; Greve and Fischl, 2009). The T1-weighted scans were non-linearly registered to the MNI 152 standard space (the Montreal Neurological Institute, Montreal, QC, Canada) using FMRIB's Non-linear Image Registration Tool. Registration parameters were estimated on non-smoothed data to transform fMRI scans into standard space. Automatic Removal Of Motion Artifacts based on Independent Component Analysis (ICA-AROMA vs0.3-beta) was used to detect and remove motion-related artifacts. ICA decomposes the data into independent components that are either noise-related or pertain to functional networks. ICA-AROMA attempts to identify noise components by investigating its temporal and spatial properties and removes these components from the data that are classified as motion-related. Registration was thereafter applied on the denoised functional data with registration as derived from non-smoothed data. As recommended, high pass temporal filtering (with a high pass filter of 150 s) was applied after denoising the fMRI data with ICA-AROMA (Pruim et al., 2015a,b; Klaassens et al., 2017, p. 311).”

##### Estimation of network connectivity

RS-fMRI networks were thereafter extracted from each individual denoised RS-fMRI dataset (12 subjects × 3 groups × 6 scans = 216 datasets) applying a dual regression analysis (Beckmann et al., 2009; Filippini et al., 2009) based on 10 predefined standard network templates as used in our previous research (Klaassens et al., 2015, p. 442): “These standard templates have previously been identified using a data-driven approach (Smith et al., 2009) and comprise the following networks: three visual networks (consisting of medial, occipital pole, and lateral visual areas), DMN (medial parietal, bilateral inferior—lateral—parietal and ventromedial frontal cortex), cerebellar network, sensorimotor network (supplementary motor area, sensorimotor cortex, and secondary somatosensory cortex), auditory network (superior temporal gyrus, Heschl's gyrus and posterior insular), executive control network (medial—frontal areas, including anterior cingulate and paracingulate) and two frontoparietal networks (frontoparietal areas left and right). In addition, time series of white matter (measured from the center of the corpus callosum) and cerebrospinal fluid (measured from the center of lateral ventricles) were included as confound regressors in this analysis to account for non-neuronal signal fluctuations (Birn, 2012). With the dual regression method, spatial maps representing voxel-to-network connectivity were estimated for each dataset separately in two stages for use in group comparisons. First, the weighted network maps were used in a spatial regression into each dataset. This stage generated 12 time series per dataset that describe the average temporal course of signal fluctuations of the 10 networks plus 2 confound regressors (cerebrospinal fluid and white matter). Next, these time series were entered in a temporal regression into the same dataset. This resulted in a spatial map per network per dataset with regression coefficients referring to the weight of each voxel being associated with the characteristic signal change of a specific network. The higher the value of the coefficient, the stronger the connectivity of this voxel with a given network. These individual statistical maps were subsequently used for higher level analysis.”

##### Higher level analysis

To investigate whether voxel wise functional connectivity with each of the 10 functional networks differed between groups, ANOVA *F*-tests were performed on four contrasts of interest (young > older adults, older > young adults, older adults > AD patients and AD patients > older adults). Networks with a significant outcome were followed by *post-hoc* unpaired two-sample *t*-tests to investigate the four contrasts separately. These tests were performed with and without correction for gray matter (GM) volume. For correction, a voxelwise partial volume estimate map of GM, as calculated from T1-weighted images with FMRIB's Automated Segmentation Tool (FAST) (Zhang et al., 2001), was added as nuisance regressor. As the results of this analysis may depend on the selection of the 10 functional networks derived from 36 healthy adults (mean age 28.5) as spatial regressors (Smith et al., 2009), we also explored a number of data driven extracted networks with Independent Component Analysis using FSL's MELODIC vs3.14. Of 70 extracted networks, the 20 networks that correlated highest with the 10 networks of Smith et al. (2009) were chosen for group analyses in order to compare these with the results of the 10 functional networks. Therefore, these 20 networks were entered in a dual regression analysis to obtain spatial connectivity maps per network per dataset followed by higher level analysis as described below.

To test for differences in connectivity between young and older adults and between AD patients and older adults across the six repeated measures per subject we used non-parametric combination (NPC) as provided by FSL's Permutation Analysis for Linear Models tool (PALM vs94-alpha; Pesarin, 1990; Winkler et al., 2014, 2016). NPC is a multivariate method that offers the possibility to combine data of separate, possibly non-independent tests, such as our repeated measures (six scans per subject), and investigate the presence of joint effects across them, in a test that has fewer assumptions and is more powerful than repeated-measurements analysis of variance (ANOVA) or multivariate analysis of variance (MANOVA). To measure these joint effects (combining the six scans per subject to one composite variable), NPC testing first performs an independent test for each repeated measure using 5,000 synchronized permutations. These tests are then combined non-parametrically via NPC using Fisher's combining function (Fisher, 1932) and the same set of synchronized permutations. A liberal mask was used to investigate voxels of gray and white matter within the MNI template, excluding voxels belonging to cerebrospinal fluid. Threshold-free cluster enhancement was applied to each independent test and after the combination, and the resulting voxelwise statistical maps were corrected for the familywise error rate using the distribution of the maximum statistic (Smith and Nichols, 2009; Winkler et al., 2014). Voxels were considered significant at *p* < 0.05, corrected.

## Results

Significant *F*-test results pointed to differences in connectivity in AD patients vs. elderly controls and in older vs. young adults for all networks, except the cerebellar network.

### Resting state connectivity without correction for GM volume

**Figure 1:**
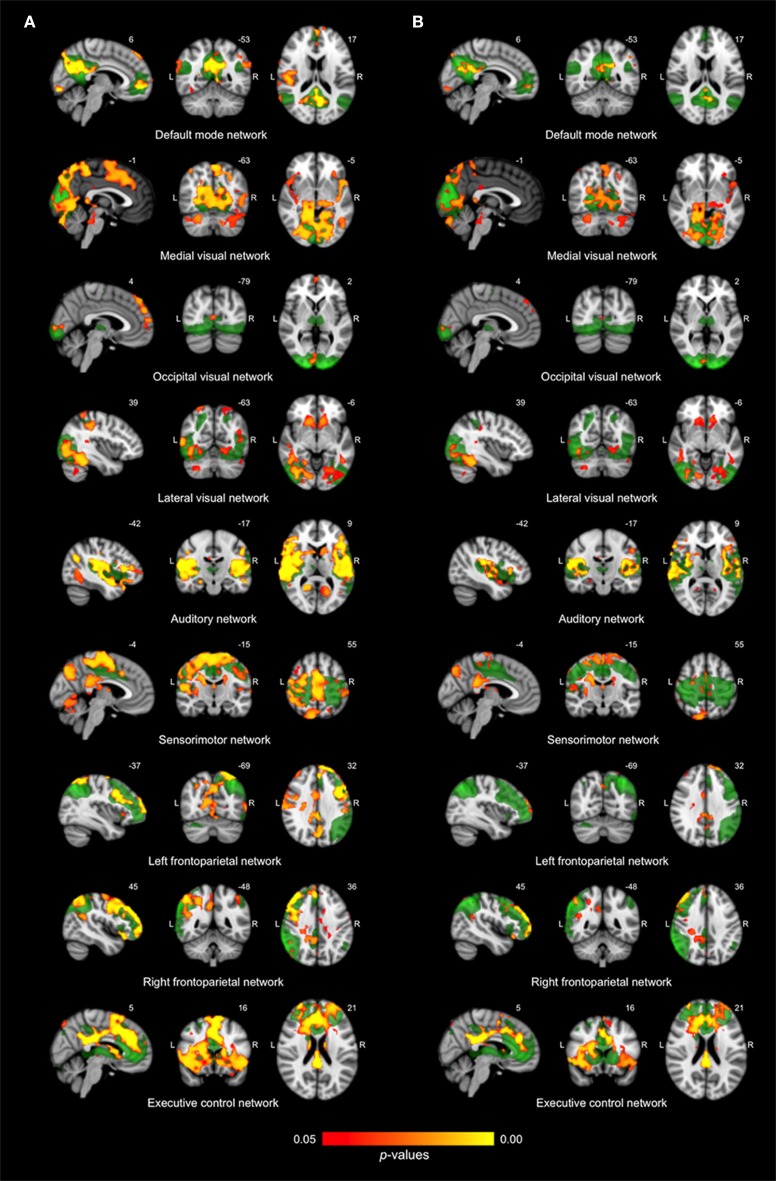
**Differences in network connectivity between young and older adults. (A)** Reduced functional connectivity in older compared to young adults between the default mode network, three visual networks, the auditory network, the sensorimotor network, the left and right frontoparietal network and the executive control network (shown in green) and regions as shown in red-yellow (at *p* < 0.05, corrected). **(B)** Reduced functional connectivity in older compared to young adults when including regional gray matter volume as regressor.

**Figure 2:**
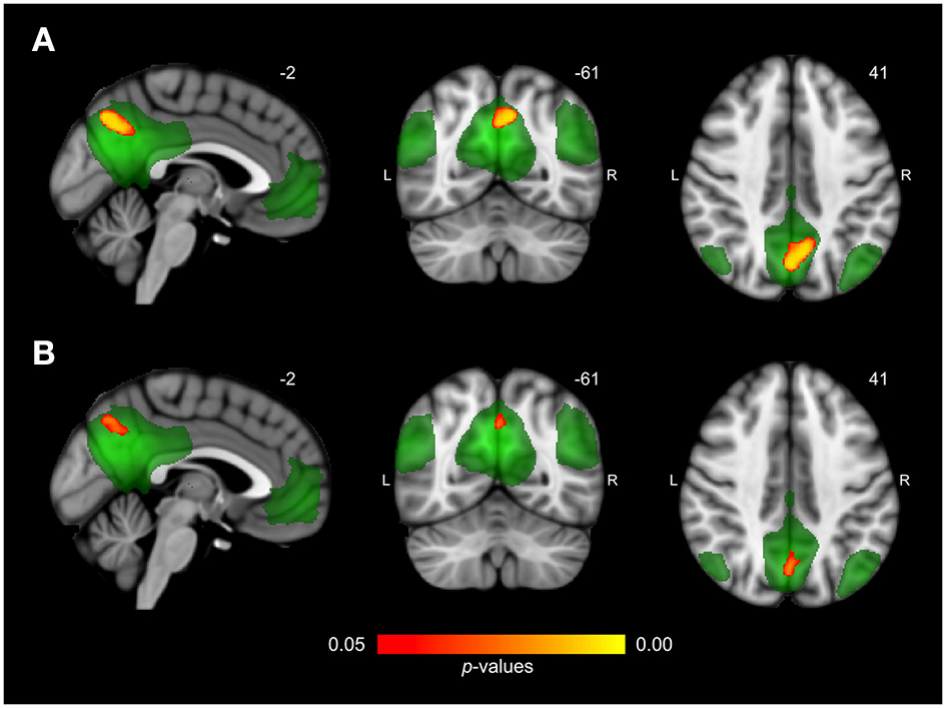
**Differences in network connectivity between AD patients and elderly controls**. **(A)** Reduced functional connectivity in AD patients compared to elderly controls between the default mode network (shown in green) and the precuneus (shown in red-yellow at *p* < 0.05, corrected). **(B)** Reduced functional connectivity in AD patients compared to elderly controls when including regional gray matter volume as regressor.

Differences in resting state functional connectivity were most apparent between young and older adults (see Figure 1A). For all functional networks, except the cerebellar network, connectivity was decreased in the older compared to the young adults, involving most cortical and subcortical regions. AD patients and elderly controls differed in connectivity with the DMN, that showed lower connectivity with the precuneus in AD patients compared to older adults (see Figure 2A). None of the networks showed higher connectivity in the older as opposed to young adults or in AD patients as opposed to the elderly controls. Specifications of effects (sizes of significant regions and peak *z*-values) are provided in Table 2. These results using 10 pre-defined networks as spatial regressors were largely similar to the results using independent component analysis to extract 70 networks from the current data, of which 20 were used as spatial regressors (see methods).

**Table 2:** **Overview of significant differences in functional connectivity without gray matter correction as estimated with threshold-free cluster enhancement (*p* < 0.05, corrected)**.

**Network**	**Contrast**	**Region (Harvard-Oxford)**		z^*^	***x***	***y***	***z***	**# voxels**
Default mode network	Older < young adults	L/R/M	Precuneus, PCC, ACC, cuneal cortex, lingual gyrus, supracalcarine cortex, lateral occipital cortex, parahippocampal gyrus, hippocampus	9.60	0	−42	20	7,814
		M	Frontal pole, frontal medial cortex, ACC, paracingulate gyrus	9.27	4	56	−6	2,600
		R	Middle and superior temporal gyrus, parietal operculum cortex, central opercular cortex, insular cortex, Heschl's gyrus, pre- and post-central gyrus	6.95	50	−20	12	2,382
		R	Lateral occipital cortex	7.04	−38	−70	54	1,436
		R	Middle and superior temporal gyrus	7.60	56	−8	−26	561
		L	Middle and superior temporal gyrus	6.97	−58	−34	−8	328
		L	Middle and inferior temporal gyrus	7.43	−58	−10	−18	299
		R	Parahippocampal gyrus, temporal fusiform cortex	5.48	20	−40	−14	154
		R	Temporal pole	5.71	50	20	−22	102
Default mode network	AD patients < controls	M	Precuneus, PCC	7.39	0	−70	44	415
Executive control network	Older < young adults	L/R/M	Frontal pole, ACC, PCC, precuneus, thalamus, putamen, SMA, post- and pre-central gyrus, temporal pole, frontal orbital cortex, superior frontal gyrus	10.90	0	−28	28	21,857
		M	Precuneus, lateral occipital cortex	6.84	8	−78	52	735
		R	Lateral occipital cortex, occipital fusiform gyrus	5.94	42	−82	4	323
		R	Cerebellum	5.65	40	−54	−36	153
		R	Pre- and postcentral gyrus, precuneus, PCC	5.48	−12	−36	46	103
Sensorimotor network	Older < young adults	L/R/M	PCC, precuneus, lingual gyrus, paracingulate gyrus, pre- and postcentral gyrus, SMA, central opercular cortex, caudate, thalamus	7.66	64	−10	42	32,668
		R	Frontal pole, middle frontal gyrus	6.18	38	46	32	442
Visual network 1	Older < young adults	L/R/M	Intracalcarine cortex, supracalcarine cortex, occipital pole, precuneus, cerebellum, PCC, pre- and post-central gyrus, brain stem, thalamus, parahippocampal gyrus, planum temporale, Heschl's gyrus, middle and inferior temporal gyrus	7.81	14	−42	−6	35,606
		R	Frontal pole	8.88	38	48	28	869
Visual network 2	Older < young adults	M	Frontal pole, paracingulate gyrus	6.75	2	56	32	1,143
		M	Occipital pole, intracalcarine cortex, lingual gyrus	7.62	8	−94	6	258
Visual network 3	Older < young adults	R	Supramarginal gyrus, pre- and postcentral gyrus, superior and middle temporal gyrus, temporal occipital fusiform cortex,	6.86	46	−32	40	8,644
		L	Supramarginal gyrus, superior and middle temporal gyrus, temporal occipital fusiform cortex, hippocampus	7.55	−18	−94	4	4,065
		L/R/M	Putamen, accumbens, frontal orbitol and medial cortex	7.35	16	22	−6	1,160
		L	Postcentral gyrus	6.62	−30	−50	72	838
Auditory network	Older < young adults	L/R/M	Heschl's gyrus, planum polare, supracalcarine cortex, caudate, putamen, hippocampus, parahippocampal gyrus, precuneus, middle and superior temporal gyrus, insular cortex, inferior and middle frontal gyrus	8.69	66	−30	20	31,540
		M	Precuneus, lateral occipital cortex	6.85	12	−76	56	1,082
		R	Superior parietal lobule, angular gyrus	7.73	46	−48	54	399
		M	PCC, ACC	7.00	8	−8	26	138
Frontoparietal network R	Older < young adults	R	Frontal pole, middle and superior frontal gyrus, precentral gyrus,	8.80	38	50	23	9,850
		R/M	Angular gyrus, superior parietal lobule, supramarginal gyrus, precuneus	8.13	48	−52	52	5,209
		L	Planum temporale, precentral gyrus, Heschl's gyrus	5.72	−58	−2	2	621
		M	Occipital fusiform gyrus, cerebellum	5.82	−14	−84	−26	549
		M	Superior parietal lobule, supramarginal gyrus	6.43	−42	−50	54	451
		M	PCC, ACC	4.98	−22	−36	36	182
		R	Middle and superior temporal gyrus	4.83	62	−26	−12	142
		R	Planum temporale	5.73	60	−20	12	136
Frontoparietal network L	Older < young adults	L/R/M	Middle, inferior and superior frontal gyrus, ACC, caudate, thalamus, pre- and postcentral gyrus	8.83	−4	34	62	17,586
		R	Pre- and postcentral gyrus	5.73	42	8	24	2,362
		M	Middle and inferior temporal gyrus	7.16	−66	−54	−2	1,460
		R	Frontal pole, middle frontal gyrus	6.65	42	48	26	1,052
		M	Frontal orbitol cortex, insular cortex	6.30	−30	20	−26	455

*L, left; R, right; M, midline; ACC, anterior cingulate cortex; PCC, posterior cingulate cortex; SMA, supplementary motor area; Voxel dimension = 2 × 2 × 2 mm (voxel volume 0.008 mL)*.

* *Standardized z-value of the uncorrected peak Fisher (NPC) statistic within regions (for regions with > 100 voxels)*.

**Figure 3:**
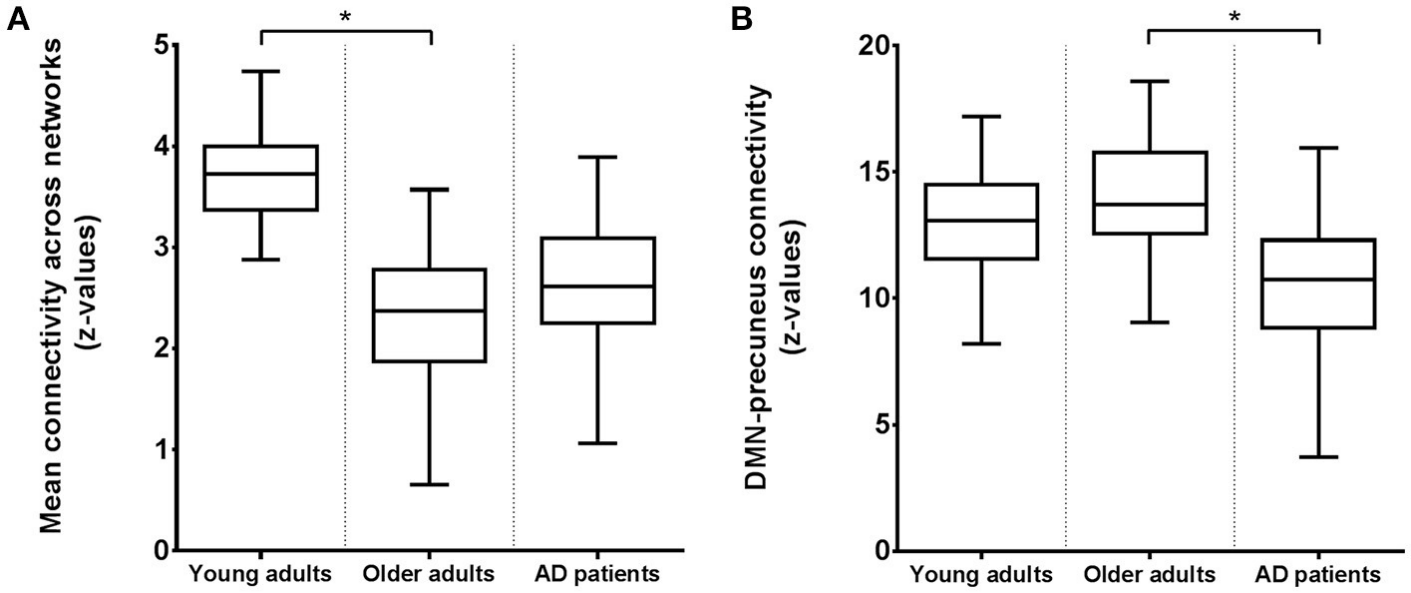
**Boxplots of the average functional connectivity (*z*-values) in young and older adults and AD patients between (A)** regions and networks as shown in Figure 1A with reduced connectivity in elderly compared to young subjects; **(B)** the precuneus and DMN as shown in Figure 2A with reduced connectivity in AD patients compared to elderly controls. Asterisks indicate a significant difference between groups (at *p* < 0.05, corrected).

Figure 3 shows connectivity for all three groups, where Figure 3A corresponds to the mean connectivity of significant voxels across all networks in Figure 1A (young vs. older adults). This illustrates that the average connectivity in these regions is significantly different between young and older adults but not between elderly controls and AD patients. Figure 3B corresponds to the mean connectivity of significant voxels for the DMN in Figure 2A (elderly controls vs. AD patients). This illustrates that the average connectivity in this region (posterior precuneus) is significantly different between AD patients and elderly controls but not between young and older adults.

### Resting state connectivity after regional correction for GM volume

After correction for regional GM volume, differences in resting state functional connectivity between young and older adults were less profound with a reduction in the number of significant voxels of 58.9% (see Figure 1B). Reduced connectivity with the same functional networks in the group of older compared to young adults mainly involved midline regions (posterior and anterior cingulate cortex, precuneus), occipital, temporal, and frontal areas. The difference between elderly controls and AD patients was more restricted after correction as well (reduction of 65.8% in the number of significant voxels) but still involved a decrease in connectivity of the DMN with the precuneus in AD patients (see Figure 2B). Specifications of effects (sizes of significant regions and peak *z*-values) are provided in Table 3.

**Table 3:** **Overview of significant differences in functional connectivity with gray matter correction as estimated with threshold-free cluster enhancement (*p* < 0.05, corrected)**.

**Network**	**Contrast**	**Region (Harvard-Oxford)**		z^*^	***x***	***y***	***z***	**# voxels**
Default mode network	Older < young adults	M	PCC, precuneus, lingual gyrus	7.92	−2	−40	20	3,214
		L	Lateral occipital cortex	8.08	−48	−58	42	244
		L	Lateral occipital cortex	6.31	−42	−64	58	113
Default mode network	AD patients < controls	M	Precuneus, PCC	7.52	0	−70	44	142
Executive control network	Older < young adults	L/R/M	Frontal pole, middle frontal gyrus, ACC, PCC, precuneus, thalamus, SMA	10.70	0	−28	28	14,548
		M	Lateral occipital cortex, precuneus	5.94	10	−78	50	161
Sensorimotor network	Older < young adults	L/R/M	PCC, precuneus, lingual gyrus, paracingulate gyrus, pre− and postcentral gyrus, SMA, central opercular cortex, caudate, thalamus	7.40	4	−38	24	12,667
		L	Postcentral gyrus	7.83	−62	−12	46	525
Visual network 1	Older < young adults	L/R/M	Precuneus, PCC, lateral occipital cortex, precentral gyrus, supramarginal gyrus, lingual gyrus, parahippocampal gyrus, hippocampus, thalamus	7.42	24	−54	−2	16,775
		L	Frontal pole	7.68	−34	50	30	664
		R	Frontal pole	8.21	36	48	28	205
Visual network 2	Older < young adults	M	Frontal pole, superior frontal gyrus	6.66	6	48	46	167
		M	Occipital pole	7.86	8	−94	6	103
Visual network 3	Older < young adults	R	Temporal occipital fusiform cortex, lateral occipital cortex, cerebellum	7.37	44	−42	−16	3,016
		L	Temporal occipital fusiform cortex, inferior temporal gyrus, cerebellum	6.18	−46	−54	−24	1,207
		L	Occipital pole	7.49	−18	−94	4	1,013
		R	Supramarginal gyrus	5.82	52	−26	32	289
		L	Subcallosal cortex, medial frontal cortex	6.66	−10	26	−6	270
		R	Cerebellum	5.74	32	−58	−42	250
		R	Frontal orbital cortex	6.27	16	24	−8	242
		L	Supramarginal gyrus	5.60	−58	−36	34	151
Auditory network	Older < young adults	R	Superior temporal gyrus, planum temporale, Heschl's gyrus, supramarginal gyrus, insular cortex, inferior frontal gyrus	7.58	66	−30	10	7,741
		L	Parietal operculum cortex, Heschl's gyrus, supramarginal gyrus, insular cortex, middle and inferior temporal gyrus	7.86	−54	−26	16	6,219
		L	Lingual gyrus, PCC, parahippocampal gyrus	5.55	−22	−62	−2	603
		R	Lingual gyrus, precuneus, PCC, parahippocampal gyrus	5.55	22	−44	−4	471
		M	Precuneus, lateral occipital cortex	6.34	0	−74	50	361
		M	PCC, ACC	7.04	8	−8	26	134
		R	Temporal occipital fusiform cortex	4.62	36	−46	−20	100
Frontoparietal network R	Older < young adults	R	Frontal pole, middle frontal gyrus	8.54	42	50	28	3,948
		R/M	Postcentral gyrus, PCC, precuneus, superior parietal lobule	5.5	36	−26	42	1,153
		R	Angular gyrus	6.56	50	−48	50	505
		R	Temporal pole, inferior frontal gyrus	8.21	54	18	−10	353
		R/M	Lateral occipital cortex, precuneus	5.71	10	−76	56	104
Frontoparietal network L	Older < young adults	M	Precuneus, PCC, caudate, thalamus	5.97	0	−62	42	1,115
		L	Frontal pole	6.74	−20	66	16	1,070
		R	Frontal pole	6.44	42	48	26	277
		R	Occipital pole	6.71	10	−92	−4	228
		L	Inferior temporal gyrus, temporal occipital fusiform cortex	6.44	−48	−56	−12	194
		M	ACC	5.91	6	2	32	134
		L	Lateral occipital cortex, superior division	7.01	−28	−74	56	122

*L, left; R, right; M, midline; ACC, anterior cingulate cortex; PCC, posterior cingulate cortex; SMA, supplementary motor area Voxel dimension = 2 × 2 × 2 mm (voxel volume 0.008 mL)*.

* *Standardized z-value of the uncorrected peak Fisher (NPC) statistic within regions (for regions with > 100 voxels)*.

## Discussion

We investigated how functional brain connectivity patterns in aging relate to connectivity as seen in AD. Brain connectivity as measured with RS-fMRI was most profoundly different between young and older adults. In contrast to the widespread disruptions in connectivity due to normal aging, the only altered network in the group of AD patients was the DMN, showing a decline in connectivity with the precuneus. This connotes that on top of reductions due to normal aging, there was an additional decrease in connectivity between the DMN and precuneus in our AD sample. A comparable effect (reduced precuneus-DMN connectivity) was found in our older adults compared to young subjects, even after GM volume control, indicating that both aging and, to a greater extent, AD compromise DMN-precuneus connectivity. The precuneus area that showed differences between groups did not exactly overlap for both comparisons. This is illustrated by Figure 3B, showing that DMN-precuneus connectivity for this specific part of the precuneus significantly differs between AD patients and older control adults but not between older and young adults. In AD patients vs. elderly controls, the effect was located more posteriorly than for the older vs. young subjects. Correspondingly, it is especially the posterior part of the precuneus that seems to be implicated in episodic memory retrieval (Cavanna and Trimble, 2006). However, considering the small sample size and possible disease specific reorganization of cortical boundaries (Sohn et al., 2015), this lack of overlap does not conclusively point to AD specific connectivity alterations.

Although there are some indications for connectivity change in frontoparietal, executive (Agosta et al., 2012), visual sensory, cerebellum/basal ganglia (Binnewijzend et al., 2012), dorsal attention, sensory-motor, control, and salience (Zhou et al., 2010; Brier et al., 2012) networks, the most consistent and frequent finding in AD is a reduction in DMN connectivity (Greicius et al., 2004; Zhang et al., 2010; Pievani et al., 2011; Hafkemeijer et al., 2012). Brier et al. (2012) showed that more networks become affected with increasing disease severity, which might declare the lack of alterations in networks beyond the DMN in our mild AD group. The relevance of the DMN in AD is explained by its core regions (precuneus, posterior cingulate cortex) being the target of β-amyloid deposition, one of the hallmarks of dementia (Buckner et al., 2005; Adriaanse et al., 2014). The precuneus comprises a central region of the DMN (Utevsky et al., 2014), with the highest metabolic response during rest (Gusnard and Raichle, 2001) and strong connections with adjacent and remote regions (Achard et al., 2006). Altered connectivity with the precuneus in AD patients has frequently been observed (Wang et al., 2006; He et al., 2007; Sheline et al., 2010; Zhou et al., 2010; Binnewijzend et al., 2012; Damoiseaux et al., 2012; Kim et al., 2013; Tahmasian et al., 2015). The precuneus seems to play a significant role in episodic memory retrieval, self-consciousness and visual-spatial imagery (Cavanna and Trimble, 2006; Zhang and Li, 2012) and structural and task-related functional MRI studies have shown its association with memory problems and visual-spatial symptoms in AD (Rombouts et al., 2005; Karas et al., 2007; Sperling et al., 2010). Involvement of the precuneus in early AD has also been demonstrated by inflated uptake of Pittsburgh compound B ([^11^C]PIB) in this area during positron emission tomography (PET), indicating increased levels of beta amyloid compared to non-demented subjects (Mintun et al., 2006). Studies that investigated pharmacological effects in AD show the importance of precuneus connectivity in AD as well. Memantine, an N-methyl-d-aspartate (NMDA) receptor antagonist and galantamine, a cholinesterase inhibitor, both used for treatment of early AD symptoms, increased resting-state functional connectivity between the DMN and precuneus in AD (Lorenzi et al., 2011; Blautzik et al., 2016), pointing to a normalizing effect of these compounds on AD symptomatology.

In contrast to the restricted DMN-precuneus disconnections in AD, aging effects on connectivity were extensive, involving multiple networks and regions. These findings indicate that functional network coherence is more sensitive to aging than AD. Reduced connectivity in the older adults was demonstrated for networks that pertain to language, attention, visual, auditory, motor and executive functioning as well as the DMN. The widespread decreases in connectivity in the older adults compared to the young group may be representative of age-related cognitive, sensory and motor decline. Hearing, vision and balance-gait problems arise and a gradual decrease in processing speed, episodic and working memory takes place during the process of normal aging (Salthouse, 1996; Li and Lindenberger, 2002; Fandakova et al., 2014). The effects for the sensorimotor and frontoparietal networks are in line with studies of Allen et al. (2011), Andrews-Hanna et al. (2007), Tomasi and Volkow (2012), and Wu et al. (2007a,b), showing an age-related decrease of connectivity between and within motor and attention networks. The cognitive function of the DMN is not fully understood, but diminished connectivity of this network is likely accompanied by a general disturbance in switching to higher-order cognitive processes as (autobiographical) episodic memory, introspection and attention (Grady et al., 2010; Mevel et al., 2011). The reduced coherence of DMN regions might reflect an inability to shift from a task-negative to a task-positive mode and hence hinder cognitive performance. This is concordant with results of Andrews-Hanna et al. (2007) and Damoiseaux et al. (2008), who demonstrated that alterations of the DMN in elderly subjects were associated with memory, executive functioning, and processing speed.

It is questionable whether group differences in connectivity are fully or partly explained by reductions in GM volume. Although exact causal mechanisms are not completely clear, connectivity alterations are possibly representative of structural atrophy (Seeley et al., 2009). A global decrease in GM has been found with advancing age, affecting frontal, parietal, temporal and occipital cortices, precuneus, anterior cingulate, insula, cerebellum, pre-, and post-central gyri (Good et al., 2001; Giorgio et al., 2010; Habes et al., 2016). It has been proposed that ignoring structural information in voxelwise analyses could bias interpretation of functional outcomes (Oakes et al., 2007), as apparent functional differences might be solely the consequence of anatomical variation. However, consistent with our outcome, it has also been demonstrated that age-related differences in functional connectivity cannot merely be explained by local decreases in GM volume (Damoiseaux et al., 2008; Glahn et al., 2010; Zhou et al., 2010; Onoda et al., 2012). When we added voxelwise GM volume maps as confound regressor to account for its possible mediating effect, a substantial portion of results (41.1%), involving equal networks, was maintained. For those areas, GM partial volume fraction is expected to be homogeneous among groups and functional effects are strong enough to persist after correction. Although the earliest atrophy in Alzheimer's disease (AD) occurs in medial temporal structures as the hippocampus (Chan et al., 2001; Matsuda, 2013), the precuneus has also been discovered as an area where atrophy appears in AD patients (Baron et al., 2001; Karas et al., 2007; Bailly et al., 2015). The observed difference between AD patients and elderly controls partly survived correction for GM volume (34.2%), suggesting that this finding is related to differences in cortical volume as well. More important, as the remaining effect on connectivity was unrelated to local structural differences, reduced DMN-precuneus connectivity might be an indicator of AD.

The small sample size (*n* = 12 per group) is an obvious limitation of our study as this reduces the power of the statistical analyses. It is possible that with a larger sample size, the DMN-precuneus connectivity change would show more overlap between the two group comparisons. However, we collected six RS-fMRI scans per subject, leading to a dataset of 72 scans per group. In addition to a gain in power, this offered us the possibility of investigating intrasubject as well as intersubject variation. The difference in effect for both group comparisons may partially be explained by higher within and between subject variance at older age and in AD (Huettel et al., 2001; Mohr and Nagel, 2010). An exploration of the average connectivity (in *z*-values) across networks and voxels per scan did not show prominent differences in connectivity variance between the three groups (young subjects: mean = 4.12, variance^between^ = 0.90 and variance^within^ = 0.86; older adults: mean = 4.37, variance^between^ = 1.63, and variance^within^ = 1.29; AD patients, mean = 4.28, variance^between^ = 0.86 and variance^within^ = 1.26), largely ruling out this possibility. Further, although all older adults were intensively screened before study participation, no information on AD-associated biomarkers was available. As alterations in brain connectivity might also be due to beta-amyloid deposition in older people without AD (Brier et al., 2014; Elman et al., 2016), the healthy elderly subjects in this study might unexpectedly include subjects in a preclinical AD stage, leading to AD- instead of age-related connectivity change.

In conclusion, differences in functional connectivity between young and older adults are more extensive than differences between AD patients and controls. We found reduced connectivity throughout the entire brain in older compared to young adults, which is potentially reflective of a normative decline in sensory, motor and cognitive function during senescence. In AD patients vs. elderly controls, the detected effect was restricted to further diminished connectivity of the DMN with the precuneus. Although the majority of these connections was associated with regional brain volume, effects were maintained for all networks after correction for GM volume. Our findings imply that posterior precuneus-DMN disconnections may act as a marker of AD pathology.

## Author contributions

JvG, SR, and JvdG: Substantial contributions to the conception or design of the work, data acquisition. JvG, SR, JvdG, and Fd: Data analysis. JvG, SR, JvdG, Fd, and CM: Interpretation of data, drafting, and critical revision of the work for important intellectual content. All authors have approved the final version of the work and agree to be accountable for all aspects of the work in ensuring that questions related to the accuracy or integrity of any part of the work are appropriately investigated and resolved.

## Funding

This study was funded by the Netherlands Initiative Brain and Cognition (NIHC), a part of the Netherlands Organization for Scientific Research (NWO) (grant number 056-13-016). SR is supported by a VICI grant from NWO (grant number 016-130-677).

### Conflict of interest statement

The authors declare that the research was conducted in the absence of any commercial or financial relationships that could be construed as a potential conflict of interest.
