# Supplementary material for: Diminished Posterior Precuneus Connectivity with the Default Mode Network Differentiates Normal Aging from Alzheimer's Disease
Source: Front Aging Neurosci. 2017 Apr 19;9:97. doi: 10.3389/fnagi.2017.00097 (PMC5395570; doi:10.3389/fnagi.2017.00097)
Supplement: Supplementary file 1 [file DataSheet1.docx]

***Supplementary Material***

**Diminished Posterior Precuneus Connectivity with the Default Mode Network Differentiates Normal Aging from Alzheimer’s Disease**

**Bernadet L. Klaassens^1,2,3,4,*^, Joop M.A. van Gerven^d^, Jeroen van der Grond^2^,**

**Frank de Vos^1,2,3^, Christiane Möller^1,2,3^ , Serge A.R.B. Rombouts^1,2,3^**

^1^Leiden University, Institute of Psychology, Leiden, the Netherlands

^2^Leiden University Medical Center, Department of Radiology, Leiden, the Netherlands

^3^Leiden University, Leiden Institute for Brain and Cognition, Leiden, the Netherlands

^4^Centre for Human Drug Research, Leiden, the Netherlands

***Corresponding author:**

Bernadet L. Klaassens MSc

Leiden University, Institute of Psychology

Unit Methodology and Statistics

PO Box 9555

2300 RB, Leiden

The Netherlands

Tel: +31 71 527 7330

Fax: +31 71 527 3761

Email: b.l.klaassens@fsw.leidenuniv.nl

All subjects performed cognitive tasks on a computerized NeuroCart^®^ test battery measuring alertness, mood and calmness (Visual Analogue Scales (VAS) Bond & Lader), vigilance and visual motor performance (Adaptive Tracking task), reaction time (Simple Reaction Time task), attention, short-term memory, psychomotor speed, task switching and inhibition (Symbol Digit Substitution Test and Stroop task), working memory (N-back task) (Bond and Lader, 1974; Borland and Nicholson, 1984; Laeng et al., 2005; Lezak, 2004; Norris, 1971; Rogers et al., 2004; Stroop, 1935; Wechsler, 1981). All repeatedly measured NeuroCart^®^ endpoints were analyzed using a mixed effects model with group, time and group by time as fixed effects, subject, subject by group and subject by time as random effects (SAS for Windows V9.1.3; SAS Institute, Inc., Cary, NC, USA). As data of the Simple Reaction Time task were not normally distributed, these data were log-transformed before analysis and back transformed after analysis. Group comparisons for the cognitive and subjective tests showed differences between the young and elderly subjects and between the elderly and AD patients for memory function, learning, attention and visuomotor skill. An overview of the results on performance tasks is provided in Supplementary Figure 1.

**Supplementary Figure 1.** Bar graphs of least squares means of performance on the NeuroCart^®^ cognitive test battery with standard error of the means as error bars. Abbreviations: YA = young adults; OA = older adults; AD = patients with Alzheimer’s disease; * = significant at *p* < 0.05; ** = significant at *p* < 0.01. Note: the N-back task for AD patients is an adapted (easier) version. It was therefore not possible to compare performance between AD patients and elderly controls.

**References**

Bond A, Lader M (1974): Use of Analog Scales in Rating Subjective Feelings. Brit J Med Psychol 47:211-218.

Borland RG, Nicholson AN (1984): Visual Motor Coordination and Dynamic Visual-Acuity. Brit J Clin Pharmacol 18:S69-S72.

Laeng B, Lag T, Brennen T (2005): Reduced stroop interference for opponent colors may be due to input factors: Evidence from individual differences and a neural network simulation. J Exp Psychol Human 31:438-452.

Lezak MD. (2004) Neuropsychological Assessment. New York. Oxford University Press.

Norris H (1971): The action of sedatives on brain stem oculomotor systems in man. Neuropharmacology 10:181-91.

Rogers MA, Kasai K, Koji M, Fukuda R, Iwanami A, Nakagome K, Fukuda M, Kato N (2004): Executive and prefrontal dysfunction in unipolar depression: a review of neuropsychological and imaging evidence. Neurosci Res 50:1-11.

Stroop JR (1935): Studies of Interference in Serial Verbal Reactions. J Exp Psychol 18:643-662.

Wechsler D (1981): The Psychometric Tradition - Developing the Wechsler Adult Intelligence Scale. Contemp Educ Psychol 6:82-85.
